# Animal Models of Human Disease 2.0

**DOI:** 10.3390/ijms252413743

**Published:** 2024-12-23

**Authors:** Sigrun Lange, Jameel M. Inal

**Affiliations:** 1 Pathobiology and Extracellular Vesicles Research Group, School of Life Sciences, University of Westminster, London W1W 6UW, UK; 2 Cell Communication in Disease Pathology, School of Human Sciences, London Metropolitan University, London N7 8DB, UK; j.inal@londonmet.ac.uk; 3 Biosciences Research Group, School of Life and Medical Sciences, University of Hertfordshire, Hatfield AL10 9EU, UK

The use of animal models is crucial for advancing translational research by identifying effective treatment targets and strategies for clinical application in human disease. This Special Topic “Animal Models of Human Disease 2.0”, called for state-of-the-art primary research and review articles, inviting global experts conducting fundamental and translational research in comparative animal models of human diseases. Submissions were invited on topics related to chronic pathologies, including autoimmune and neurodegenerative diseases, cancer, acute injury, inflammatory and infectious diseases, and regenerative medicine. The five participating MDPI journals were *Biomedicines*, *Cells*, *Current Issues in Molecular Biology*, *Genes*, and the *International Journal of Molecular Sciences*. A total of twenty-two papers were published, including two comprehensive reviews on the use of diverse animal models to advance translational research and twenty original research papers using more common and conventional laboratory animal models (mice, rats, rabbits) or other less frequent comparative models (dogs and plateau pikas). Findings reported in this Special Topic provide significant new information on fundamental pathobiological mechanisms and clinical markers of chronic and acute human pathologies and investigate exposome effects on human health. Research topics included nervous system, liver, lung, and cardiac diseases, the gut microbiome, host–pathogen interactions, antibiotic resistance, pathological effects and/or therapeutic potential of selected drugs and compounds, mitochondrial-mediated mechanisms, and hypoxia-mediated pathways. Topics of regenerative medicine discussed the use of stem cells and gene-edited animal models. Research on exposome-related factors included blue light irradiation and microgravity, highlighting risks to human health regarding the overuse of smart digital devices and future long-term spaceflight missions.

The use of animal models is crucial for advancing translational research by identifying and validating effective treatment targets and strategies for clinical application in human disease [1,2]. Murine models are frequently used in laboratory settings for a wide range of disease modelling, including gene editing, and can be used to study cancer, infection, and cardiovascular, metabolic, rare, neurodegenerative, and autoimmune diseases [3,4,5,6,7,8]. For some pathologies and in further translational and preclinical studies, larger animal models closer to human physiology and anatomy may be desirable, including pigs, sheep, dogs, and nonhuman primates [9,10,11,12,13,14,15,16,17]. In addition, animals with specific characteristics which aid the study of certain organ systems may be feasible, such as *Octopus vulgaris* for visual, sensory, and nervous system research [18,19,20,21] or piglets for pediatric heart transplantation studies [22], depending on the 3Rs and utilitarian ethics. Various species lower in the phylogeny tree, including fruit flies (*Drosophila melanogaster*), nematodes (*Caenorhabditis elegans*), amphibians (*Xenopus laevis*), and fish (zebrafish, *Dano rerio*), are also common laboratory animal models for molecular and cellular mechanistic studies and drug-screening for human disease and precision medicine [23,24,25,26,27,28,29]. Furthermore, non-traditional animal models displaying unusual immune and metabolic characteristics, such as resistance to hypoxia, infection, cancer, and longevity, are of interest, as these can provide novel insights into molecular pathways and identify new treatment targets to improve human health, as well as revealing mechanisms for survival in extreme environments. Examples of such comparative and/or wild animal species include naked mole-rats [30,31], plateau pikas [32,33], tenrecs [34], reptiles [35,36,37,38], camelids [39,40,41], sharks [42,43,44], Pinnipeds (seals) and Cetaceans (whales, and dolphins) [45,46,47,48,49,50]. In addition, animals displaying unusual regenerative capacities, such as planaria, annelids, and axolotls [51,52,53,54,55,56,57,58,59], as well as embryonic chickens (*Gallus gallus*) for nervous system regeneration [60,61,62,63,64] and fish and amphibians for cardiac regeneration [65], can provide important information for regenerative medicine research. The ethical implications of the use of the most appropriate animal model(s) for each case must be critically evaluated and adhere to the 3Rs (Replacement, Reduction, and Refinement) [1,66,67]. The availability of the given animal model, including using archived samples or carrying out functional in vivo, ex vivo, in vitro, or computational modelling, must also be considered from a practical viewpoint.

This Special Topic “Animal Models of Human Disease 2.0” (https://www.mdpi.com/topics/4Q38Y4392K) (accessed on 30 November 2024), called for state-of-the-art primary research and review articles, inviting global experts conducting fundamental and translational research in comparative animal models relevant to human diseases to contribute. Submissions were invited to cover topics on chronic pathologies, including autoimmune and neurodegenerative diseases, cancer, acute injury, inflammatory and infectious diseases, and regenerative medicine. The five participating MDPI journals were *Biomedicines*, *Cells*, *Current Issues in Molecular Biology*, *Genes*, and the *International Journal of Molecular Sciences*. In total, twenty-two papers were published, including two comprehensive reviews on the use of diverse animal models to advance translational research and twenty original research papers using more common and conventional animal models such as mice, rats, and rabbits or other less frequent comparative models such as dogs and plateau pikas. The key findings of the published contributions to this Special Topic are briefly summarized below.

In Contribution 1, Chen and colleagues used rats and plateau pikas (*Ochotona curzoniae*), a high-altitude-adapted burrowing mammal, to assess the protective effects of the host’s intestinal microbiota in mitigating hypoxic damage following chronic hypoxic injury. Gut microbiota from plateau pikas were transplanted into rats that were exposed to hypoxia challenge. Beneficial effects were observed on inflammatory pathways, proposing new approaches to treat hypoxic pulmonary hypertension.

In Contribution 2, Kraus et al. used phenobarbital to develop a fast animal model of metabolic dysfunction associated with steatohepatitis (MASH-cirrhosis) in rats, reflecting the human disease. The rats developed advanced disease signs characterized by portal hypertension, blood biochemistry, hepatic ballooning, steatosis, inflammation, and fibrosis induced by phenobarbital. Their study provides a new animal model of MASH-cirrhosis that is of value to the wider research community.

In Contribution 3, Li and co-workers studied cold-induced immunosuppression in a mouse model and tested the therapeutic potential of traditional herbal medicine. The authors used the root extract from *Salvia miltiorrhiza*, assessing its active compound tanshinone IIA, showing the effects of plasma IgG and improved bacterial clearance. Their study lays the foundation for future applications of *Salvia miltiorrhiza* bioactive compounds in mitigating cold-induced immunosuppression.

In Contribution 4, Park et al. assessed the therapeutic effects of tadalafil, a phosphodiesterase type 5 (PDE-5) inhibitor, on the endothelium in a rabbit model of subarachnoid hemorrhage. The findings indicated that tadalafil indirectly prevents endothelial cell death and shows neuroprotective properties. Their study provides a basis for further investigation into apoptosis-related proteins related to tadalafil application.

Contribution 5 by Tahimic and colleagues used rat models to study the effects of microgravity on gene regulation in immunity and cardiovascular disease, including oxidative stress. Findings indicated sex-dependent changes in oxidative damage and increased inflammation in response to microgravity exposure. This paper contributes to the growing body of research on human health concerning long-term space missions and is important for future space medicine.

Contribution 6 by Nawaz and colleagues examined diabetic cardiomyopathy and cardiac remodelling in a rat model, assessing the therapeutic potential of Phoenix Dactylifera. The findings indicate cardioprotective effects through the regulation of metabolic signalling and glucolipid balance. Their study introduces a new compound for the treatment of diabetes-associated heart failure.

In Contribution 7, Fan et al. used a rat model of ischemic stroke to assess the potential of the flavonoid compound, Luteolin-7-O-β-d-glucuronide (LGU), as a neuroprotective agent. LGU was found to attenuate cerebral injury due to ischemia/reperfusion by improving the permeability of the blood–brain barrier, with molecular targets including S100B, tight junction proteins, and metalloproteinases.

In Contribution 8, Bose and co-workers created new, humanized mouse models of Gulf War Illness (GWI) to study host gut microbiome–immune interactions. The group showed that their model significantly altered gut microbiomes and modified cytokine profiles when treated with Gulf War chemicals, and these were similar to reported microbiomes in Veterans. Their study provides a new in vivo model for gut–immune interactions in GWI.

Contribution 9 by Ray et al. used a mitochondrial aldehyde dehydrogenase-2 (ALDH2) knockout mouse model showing its neuroprotective role in binge alcohol-induced brain damage, with involvement of the gut–brain axis. Their study provides a new potential target for attenuating alcohol-induced organ and tissue injury.

In Contribution 10, Hiramoto and colleagues used a mouse model to assess the negative effects of long-term blue light exposure on memory and learning ability, identifying changes in inflammatory pathways. The findings provide important insights into the health implications relating to long-term exposure to blue light, including from digital smart devices.

Contribution 11 by Munalisa and colleagues used a mouse model to assess stress-induced inflammatory responses in gastrointestinal injury. Their study used restraint stress to investigate neutrophil inflammation and showed that pharmacological NETosis inhibition protected the gastrointestinal tissue from stress-induced inflammation. It also highlights a new target in the management of gastrointestinal injuries caused by physiological stress.

In Contribution 12, Seol and co-workers developed guidelines on optimized embryo collection from rat models for gene editing approaches using the CRISPR/Cas9 system. Their study proved the efficiency of these guidelines by generating a fukutin knockout rat model, constructing the first muscular dystrophy disease rat model using the CRISPR/CAS9 system. Their study contributes a new method for gene-editing rats for application in human disease modelling.

Contribution 13 by Vacca et al. used a mouse model of intellectual disabilities in Duchenne muscular dystrophy, using adeno-associated virus administrations through intra-cardiac or intra-cerebroventricular injections. This proof-of-concept study was tested in young and adult mice and contributes to developing effective gene therapy approaches for cognitive disorders.

In Contribution 14, Watanabe and colleagues developed a new knockout mouse nephropathy model, assessing Adriamycin-induced podocyte injury. Several new genes with roles in kidney damage were identified, paving the way for new methods for early diagnosis and treatment of kidney disease. This study provides a new in vivo tool for research in chronic kidney disease.

Contribution 15 by Wen and collaborators developed a rat model to study methods to treat human patients with prolapse and hemorrhoids. This model helped identify new approaches to decrease inflammation and fibrosis in anal stenosis and is of clinical relevance.

In Contribution 16, Wang et al. used a mouse model to assess the therapeutic potential of a new aptamer in coronoviral infection-induced acute lung injury. Their study highlights Apta-1 as a therapeutic agent, protecting pulmonary endothelial integrity and reducing both infection-induced hemorrhage and systemic inflammation in coronaviral infection.

Contribution 17 by Chaklai and colleagues used mouse models with mutations linked to Parkinson’s disease and Gaucher disease (A53T and A53T-L444P) to assess the protective roles of the gut microbiome in response to exposure to paraquat, dextran sulfate sodium, and radiation. Findings indicate important roles for the gut microbiome in mediating the impacts of environmental exposures or genetic mutations on cognition and behaviour. Using a combination of colitis-induced, herbicide-exposed, and neurodegenerative mouse models, this study contributes to the increasing body of research on exposome effects and the gut–brain axis in neurological diseases.

In Contribution 18, Murgiano and collaborators identified a frameshift variant in adenosine monophosphate deaminase related to retinopathy and tremors in dell’Etna dogs. The findings reported on this oculo-neurological syndrome add to the spectrum of known neurological manifestations associated with human adenosine monophosphate deaminase variants. Their study highlights larger animal models‘ important role in understanding diverse neurological disorders.

Contribution 19 by Trivedi et al. reports the effects of prolonged antibiotic use on renal fibrosis-like pathology in a preclinical mouse model of Gulf War syndrome. The findings identify roles for TGF-beta and microRNA-21 mediated pathways in gut dysbiosis observed in Gulf War veterans. Their study adds to the current understanding of molecular mechanisms involved in mediating the complex functions of the gut microbiome in renal pathology in response to long-term antibiotic treatment.

Contribution 20 by Fioretti et al. generated a mouse model of freeze-injured skeletal muscle to mimic traumatic muscle injury and assessed the pro-regenerative potential of amniotic stem cell application, including via macrophage-related responses. Their study contributes an important new model for regenerative medicine research relating to stem cell transplantation in severe traumatic muscle injuries.

Contribution 21 by Norazaman and colleagues is a comprehensive review of animal models for studying bone health in type-2 diabetes mellitus (T2DM) and obesity. The authors further discuss the literature on the inter-relationship between diabetes, obesity, and bone loss. The key animal species reviewed were mice and rats, and the models discussed were chemical-induced, genetically modified, monogenic or polygenic, dietary-induced, and leptin-receptor-related. Their review is important for increasing the understanding of bone health in co-morbidities and age-related and chronic conditions.

In Contribution 22, Giusti and collaborators review adult and embryonal animal models for xenografting, with an emphasis on musculoskeletal sarcomas. More traditional xenograft models of mice, chick (chorioallantoic membrane), and zebrafish embryos are discussed, and how these can be used to model rare and heterogeneous sarcomas. Their review highlights the importance of using diverse animal models for boosting translational research.

In conclusion, this Special Topic covers translatable and clinically relevant findings from various comparative animal models of human disease. The diversity of the studies highlights the importance of using the most appropriate model to study fundamental disease pathways and validate druggable targets in different pathologies. In addition, the common use of mouse and rat models in laboratory settings is noted in 17 out of 20 papers in this Special Topic, while one study used rabbit, one used dog, and one used plateau pika alongside rat. The two reviews furthermore discussed the use of rat and mouse models alongside zebrafish and chick models, including at embryonic stages, for different disease studies.

Animal models will remain key critical tools to advance biological and medical research. The increasing implementation of the 3Rs and ethical considerations [66,68] is, in addition, accelerating the development of sophisticated gene editing and cellular tools, including induced pluripotent stem cells (iPSCs), bioprint models, organs-on-chips, 3D in vitro assays, organoids, and microfluidics systems [69,70,71,72]. Furthermore, developing cell lines from a wider range of organisms and animals can meet the need for a more diverse pool of model species [73,74,75,76]. A combinatory approach of in vivo, ex vivo, in vitro, and in silico methods will continue to further the current understanding of disease processes to develop improved therapeutic strategies. The findings reported in this Special Topic provide significant new information on fundamental pathobiological mechanisms and clinical markers of chronic and acute human pathologies and exposome effects on human health, not only on Earth but also in Space.
